# Relevant Strength Parameters to Allow Return to Running after Primary Anterior Cruciate Ligament Reconstruction with Hamstring Tendon Autograft

**DOI:** 10.3390/ijerph19148245

**Published:** 2022-07-06

**Authors:** Jérôme Grondin, Vincent Crenn, Marie Gernigon, Yonis Quinette, Bastien Louguet, Pierre Menu, Alban Fouasson-Chailloux, Marc Dauty

**Affiliations:** 1 Service de Médecine Physique et Réadapatation Locomotrice et Respiratoire, CHU Nantes, Nantes Université, 44093 Nantes, France; jerome.grondin@chu-nantes.fr (J.G.); bastien.louguet@chu-nantes.fr (B.L.); pierre.menu@chu-nantes.fr (P.M.); marc.dauty@chu-nantes.fr (M.D.); 2 Service de Médecine du Sport, CHU Nantes, Nantes Université, 44093 Nantes, France; 3 Clinique Chirurgicale Orthopédique et Traumatologique, CHU Nantes, Nantes Université, 44000 Nantes, France; vincent.crenn@chu-nantes.fr (V.C.); yonis.quinette@chu-nantes.fr (Y.Q.); 4 Complexité, Innovation, Activités Motrices et Sportives—CIAMS, Université Paris-Saclay, CEDEX, 91405 Orsay, France; marie.gernigon@universite-paris-saclay.fr; 5 Complexité, Innovation, Activités Motrices et Sportives—CIAMS, Université d’Orléans, 45067 Orléans, France; 6 IRMS—Institut Régional de Médecine du Sport, 44093 Nantes, France; 7 Inserm UMR 1229, Regenerative Medicine and Skeleton, RMeS, Nantes Université, 44042 Nantes, France

**Keywords:** knee, ACL reconstruction, sport, isokinetic, running

## Abstract

After anterior cruciate ligament reconstruction (ACLR), a progressive process is followed from rehabilitation to the return to sport including a crucial step known as the return to running. Return to running (RTR) can be predicted by an isokinetic knee strength assessment at 4 months post-surgery. All patients who had primarily undergone ACLR with a hamstring autograft procedure between 2010 and 2020 were included in this study. Four months after surgery, patients were evaluated using an isokinetic knee strength test. Patients were monitored until the 6th month post-surgery to see if they had returned to running. Comparisons were carried out between the two groups—the RTR and the no-RTR. A multivariate logistic regression analysis was used to predict the RTR status from explicative parameters. Receiver Operating Characteristic (ROC) curves were established to identify cutoffs with their characteristics. A total of 413 patients were included and 63.2% returned to running at 4 months post-surgery. The mean Lysholm score, knee complication rate, and isokinetic parameters were statistically different between both groups. Using a multivariate logistic regression model and ROC curves, the best isokinetic parameter to assist with the decision to allow an RTR was the quadriceps limb symmetry index at 60°/s with a cutoff of 65%. The hamstring LSI at 180°/s could be added (cutoff of 80%) to slightly increase the prediction of an RTR. Quadriceps strength normalized to body weight at 60°/s is a useful parameter (cutoff: 1.60 Nm/kg) but measurements on both sides are necessary. Isokinetic parameters are objective parameters to allow a return to running at 4 months after ACLR with a hamstring procedure.

## 1. Introduction

Nearly 300,000 anterior cruciate ligament reconstructions (ACLR) are performed in the United States each year [1]. The anterior cruciate ligament (ACL) is an intra-articular and extrasynovial structure that acts to control anterior translation and rotational movements of the femur on a fixed tibia. It runs inferiorly, medially, and anteriorly from its insertion on the lateral wall of the femoral intercondylar notch to its termination in a fovea anterior to the tibial eminence [2,3]. Anterior cruciate ligament rupture is a frequent injury in pivoting sports such as soccer, basketball, and handball, and implies likely multi-planar (combined sagittal, frontal, and transverse) mechanisms [4,5]. Around 80% of people return to sport but only 65% return to their pre-injury level and 55% return to a competitive sport level [6]. A return to the previous level of sport after ACLR remains a crucial topic for patients, whereas avoiding re-injuries, pain, and swelling are priorities for clinicians [7]. For example, patients who return to sport without fulfilling prior simple functional criteria such as a hop-test or running test are exposed to a four times increased risk of re-rupture [8]. There is a clinical need for structuring rehabilitation and reathletization protocols with valid criteria in order to minimize the risks of complications such as iterative ruptures.

To return to the expected level of performance, a progressive step-by-step continuum of rehabilitation is described including the return to running (RTR) [7]. Running implies increased knee joint work from 2 to 5 times compared to walking [9] but it does not imply the same increase in frontal (varus/valgus) and axial (internal/external rotation) moments and kinematics, as in side-cutting activities [10,11]. In patients who sustained ACLR, there is a strength deficit of the quadriceps and the hamstring [12] that significantly alters gait patterns with decreased knee angles and knee extension moments in the stance phase while walking and even more while running [13]. So, allowing an RTR requires valid criteria including strength evaluation. In a recent review, 99% of the studies reported time-based criteria for an RTR with a median of 12 post-operative weeks, and only 18% of them reported using additional criteria, which were either clinical (range of motion, effusion, pain), strength-based with isometric or isokinetic criteria, or performance-based [14].

A quadriceps limb symmetry Index (Q-LSI) > 70% is the most cited isokinetic cutoff parameter in literature without scientific proof [14]. It has recently been shown that a Q-LSI at 60°/s > 60% permitted more patients to return to running without sustaining a more iterative rupture [15]. However, this Q-LSI > 60% cutoff was chosen according to experience and an analysis using the Youden Index may clarify it. Furthermore, in this last study, the authors simultaneously included patients with Bone–Patellar Tendon–Bone (BPTB) and hamstring autograft procedures that led to different strength deficits: quadriceps strength was more impaired after BPTB and hamstring strength was more impaired after hamstring procedure [12]. There is also evidence for other isokinetic parameters of interest such as quadriceps strength reported to body weight (QS/BW) [16].

Hence, we conducted a study to identify the most relevant and accurate isokinetic strength parameters, while considering other explanatory parameters, to allow a return to running in a population of patients who sustained ACLR with a hamstring autograft procedure. Our main hypothesis was that a significant difference in strength measures between patients who returned to running and those who did not would provide reliable isokinetic strength cutoffs with good parameters (sensitivity, specificity, area under the curve). The clinical purpose of this work was to optimize the return to running, thereby the return to the expected level of performance, and to reduce the risk of re-injury.

## 2. Materials and Methods

### 2.1. Participants

We performed a retrospective analysis based on a prospective cohort of patients who underwent ACLR between 2010 and 2020. We assessed all the patients referred to the Sports Medicine Department of a University Hospital for their eligibility to partake in an isokinetic evaluation after ACLR. Patients were included at the time of their first isokinetic test, which was planned for around 4 months after surgery.

Inclusion criteria were patients who were over 18 years old and less than 45 years old, who had sustained primary ACLR, who had undergone a hamstring autograft arthroscopic procedure, who had undergone at least 2 testing sessions including clinical and isokinetic evaluations at 4 and 6 months post-operatively, and who aimed to return to running. Surgical meniscal repairs or meniscectomies and tenodesis procedures associated with ACLR were included.

Exclusion criteria were ACL revision, other ligament reconstructions such as posterior cruciate ligament or collateral ligaments, patients who had already returned to running before our evaluation, and patients whose first isokinetic test was performed more than 150 days after the surgery. Isokinetic evaluation was delayed if the patient suffered from knee swelling, knee locking, extension loss > 15°, or limping.

In this interventional study, necessary processes were performed with the “Direction de la Recherche Clinique” (DRC) of the University Hospital and ethical approval was obtained from the local committee of ethics “*Groupe Nantais d’Ethique dans le Domaine de la Santé*” (GNEDS) on 20 May 2020. The database was anonymized. All patients gave their written consent to participate in the study without receiving any financial compensation.

### 2.2. Surgical Procedure

Surgical procedures were performed in a University Hospital and in four clinics by several experienced surgeons. ACLR was performed on a daily basis with standardized arthroscopic procedures. First, a diagnostic arthroscopy was performed, including a meniscal tear evaluation that could lead to meniscal surgery, depending on the type and location of the meniscal tear. Then, a four-strand hamstring tendon autograft (semitendinosus and gracilis tendon) was harvested through a longitudinal incision over the pes anserinus insertion. The graft had a diameter of 7 to 9 mm. Tibial and femoral tunnels were drilled with out–in or in–out procedures and hamstring autograft was fixed using an endobutton for femoral fixation and screws for tibial fixation.

### 2.3. Rehabilitation Protocol 

The early rehabilitation program included the following [15]:

- Treatment of knee-swelling, edema, and pain by compression, icing, and non-steroidal anti-inflammatory drugs until complete swelling and heat resorption.
- Limitation of standing and walking in order to limit knee-swelling and pain.
- Full weight-bearing allowed with crutches.
- Use of knee brace until full active knee extension was achieved.
- Progressive recovery of knee range of motion with special care for preserving full active knee extension throughout the process.

The mid-term rehabilitation program included the following:

- Stop using crutches when the gait is well-balanced after proprioception exercises.
- Possibility of driving a car or returning to professional sedentary activities when walking without crutches was achieved.
- Return to cycling when knee range of motion was at least 0 to 120 degrees of knee flexion. A progressive protocol was given to all patients: cycling was practiced 3 times a week from 15 min to 90 min sessions over a 2-month period until the first isokinetic test.

### 2.4. Isokinetic Evaluation Procedure

All patients underwent a clinical evaluation to ensure knee stability and the absence of swelling before isokinetic strength assessment. After a 10-min cyclo-ergometer warm-up, isokinetic strength was evaluated with a Cybex Norm dynamometer (Lumex Inc., Ronkonkoma, NY, USA). The isokinetic procedure was performed as previously reported [17] and conducted by the same physician (MD). After instruction, the knees were evaluated from 0° to 100° of flexion, beginning with the non-operated side and with verbal encouragement and visual feedback. After familiarization with the isokinetic movement with mild strength movements, 3 repetitions were performed in concentric mode at 60°/s followed by 5 concentric repetitions at 180°/s, each of them performed with maximal strength. Quadriceps and hamstring peak torque was normalized to body weight (QS/BW and HS/BW, respectively). Limb symmetry indexes (Q-LSI and H-LSI) were calculated as follows: (peak torque of operated side/peak torque of non-operated side) × 100. Hamstring-to-quadriceps ratios (H/Q) were also calculated for the two angular speeds and for the two sides. The different isokinetic parameters were considered reliable to be used for knee strength evaluation. In healthy active individuals, Q-LSI showed an intra-class correlation of 0.78 (95% confidence interval [CI]: 0.59–0.90) and QS/BW at 60°/s showed an intra-class correlation of 0.98 (95% CI: 0.95–0.99) [18].

### 2.5. Follow-Up

The first visit was performed at 4 months post-surgery with demographic characteristics collection, clinical examination, and an isokinetic test procedure. The level of sport was scored according to the Tegner score [19] and knee function was reported using the Lysholm score [19].

Complications such as anterior and posterior knee pain, arthrofibrosis, infection, or swelling were collected. Six months after ACLR, information on the patient’s RTR was collected. Return to running was considered successful if the patient had performed at least 50% of the running program provided after the testing session at 4 months. In case of non-compliance with at least 50% of the instructions, the return to running was considered unsuccessful (NRTR). For the patients initially not allowed to run, an RTR was considered if they had declared running twice a week prior to 6 months post-surgery.

### 2.6. Decision-Making Process to Allow a Return to Running

There is sufficient proof in the literature to apply isokinetic criteria in the decision process [14,15,16]. Once the isokinetic test at 4 months was performed, we deemed it unethical to permit an RTR regardless of isokinetic parameters. Hence, we used the following decision-making process based on Q-LSI calculated at 60°/s with a cutoff of 60% to authorize or not a return to running:

- If the Q-LSI at 60°/s was ≥60%, an RTR was allowed at moderate intensity (70% of maximum heart rate) 3 times a week from 15 to 30 min continuous sessions in the first month. In the second month, 3 sessions were proposed: 2 sessions of 1 to 2 min interval training at up to 85% of maximal heart rate, and one session with 10 × 50 to 100 m progressive acceleration. A written program was given to the participants and they were encouraged to buy a heart rate monitor to self-monitor their running intensity.
- If the Q-LSI was <60% but >50%, an RTR was not allowed and only cycling was prescribed. If the Q-LSI was <50%, only swimming, including breaststroke, was allowed. Physiotherapy care was prescribed only if the total knee range of motion was not achieved.

### 2.7. Statistical Analyses

Statistical analyses were performed using the software SPSS 23.0 IBM Corp, Armonk, NY, USA. Quantitative data were presented as mean and standard deviations and qualitative data as frequency. The normal distribution of the data was verified by the Shapiro–Wilk test. Comparisons of demographic characteristics between the two populations (RTR and NRTR) were performed using t-tests for quantitative variables and χ^2^ for qualitative variables. First, the associations between the dependent (RTR) and independent variables were analyzed using univariate regression. Second, a multivariate logistic regression model with forward selection (Wald) was performed, including variables with a *p* value < 0.10, to identify independent predictors of a return to running after a hamstring procedure [20,21]. Two models were built, taking into account either the isokinetic parameters of the patient as a unit (LSI) or the isokinetic parameters of the limbs as a unit (muscle strength concentric peak torque to body weight, H/Q ratios). Because of the inclusion of continuous and categorical variables, the ORs were estimated as the exponential of the coefficient B of the logistic regression [5]. The Hosmer–Lemershow test was used to describe if the data fitted the model well. The R-squares of Cox–Snell and Nagelkerke (% of the variance explained by the predictors) were used to find out if the model was well-adjusted. The Receiver Operating Characteristic (ROC) curves were established to determine the sensitivity and specificity of continuous variables included in the models. Their areas were interpreted as excellent (>0.9), good (0.8–0.9), fair (0.7–0.8), poor (0.6–0.7), or failed (0.5–0.6) [22]. Youden’s index was used in conjunction with ROC analysis to determine the optimum cutoff of numeric predictor parameters [23,24]. The cutoff was chosen for the value of the test that gave equal weight to false-positive and false-negative values. *p* < 0.05 was considered significant.

## 3. Results

**Figure 1:**
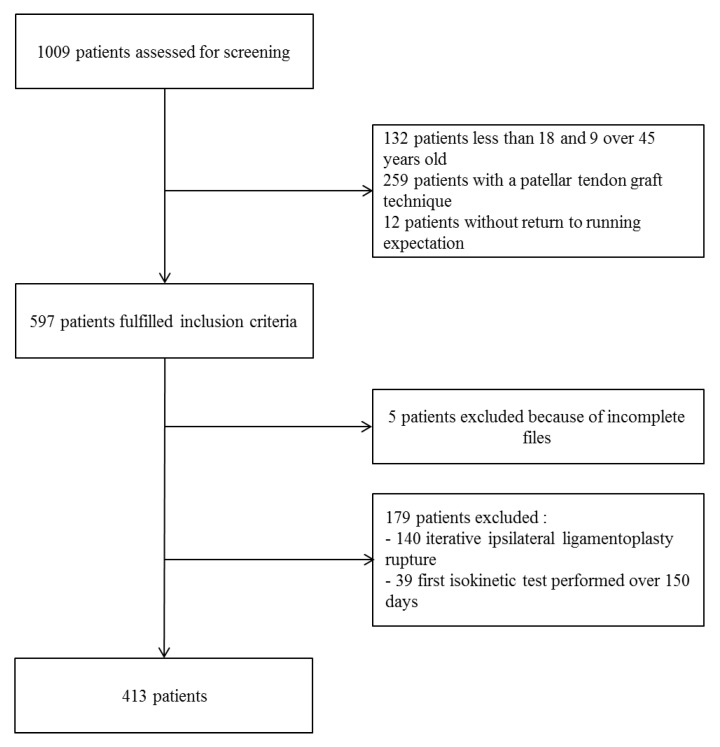
Flowchart of the included participants.

Four hundred and thirteen patients were eventually included with a mean age of 26.2 ± 6 years (Figure 1). Patients were mainly male (*n* = 290, 70.2%) and all of them had sustained a hamstring tendon autograft procedure. Before the injury, 28 patients (6.8%) were professional athletes; 39 (9.4%) were professionals in various areas of sport, such as trainers; and 71 patients (17.2%) were students. All patients practiced sport: 187 practiced soccer (45.3%), 89 basketball (21.5%), 52 handball (12.6%), 19 skiing (4.6%), 14 rugby (3.4%), and the others—52 patients (12.6%)—practiced other sports. The pre-injury Tegner Activity Scale was level 10 for 23 patients (5.6%), level 9 for 41 patients (9.9%), level 8 for 99 patients (24.0%), level 7 for 132 patients (32%), level 6 for 76 patients (18.4%), and level 5 for 42 patients (10.1%). The mean delay from surgery to the first isokinetic test was 124.9 ± 20.2 days.

### 3.1. Comparison of the Return-to-Running and the No-Return-to-Running Populations

Two hundred and sixty-one patients (63.2%) had returned to running and 152 (36.8%) had not at 6 months after ACLR. The demographic characteristics were not statistically different between the RTR and NRTR groups (Table 1). The mean Lysholm score was significantly higher in the RTR group. The quadriceps peak torque and hamstring peak torque normalized to body weight (QS/BW and HS/BW) at 60 and 180°/s on the operated limb were significantly higher in the RTR group, but no statistical difference was found on the contralateral limb. The limb symmetry indexes (Q-LSI and H-LSI) at 60 and 180°/s were significantly higher in the RTR group. The hamstring-to-quadriceps ratios at (H/Q) 60° and 180°/s were lower in the RTR group (Table 2). Complications occurred in 33.7% of the patients. The total complication rate was significantly lower in the RTR group than in the NRTR group (21.4% and 54.6%). The proportion of patients with arthrofibrosis (2.3% vs. 22.4%) and anterior knee pain (6.5% vs. 17.1%) was lower among patients who had returned to running (Table 3). No graft tears were reported in either of the populations during a return to running or not.

### 3.2. Univariate Analysis

From the univariate analysis, the Lysholm score, knee complications, and all the isokinetic parameters (LSI and strength reported to weight) were potentially relevant except for the H/Q on contralateral limb ratio. Age, sex, weight, height, delays in ACL tear surgery, delays in surgery-isokinetic tests, meniscal procedures, and pre-injury Tegner Activity Scales were not relevant to be included in the multivariate analysis.

### 3.3. Multivariate Analysis: Wald Logistic Regression

#### 3.3.1. First Model Taking into Account Patients as Unit (Limb Symmetry Indexes)

**Figure 2:**
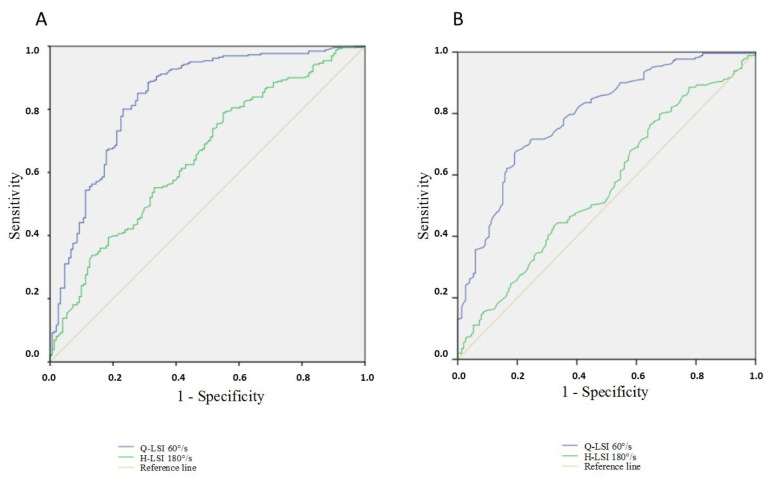
(**A**) ROC curve of Q-LSI at 60°/s and H-LSI at 180°/s; (**B**) ROC curve of QS/BW (operated limb) and QS/BW at 60°/s (contralateral limb). Q-LSI: Quadriceps Limb Symmetry Index; H-LSI: Hamstring Limb Symmetry Index. QS/BW: Quadriceps Strength peak torque to Body Weight.

The best model to predict an RTR after a hamstring procedure included the Q-LSI 60°/s and H-LSI 180°/s after the exclusion of the Lysholm score and knee complications. The percentage of correct classifications by hazard was 63.3% and the prevision by the model in step 1 was 81.0% with the Q-LSI at 60°/s and in step 2 it was 81.3% with the H-LSI at 180°/s (Table 4). The ROC curve area for the Q-LSI at 60°/s was 0.837 (95CI%: 0.795–0.880) and the sensitivity and specificity were, respectively, 78.9% and 76.8% if the cutoff of the Q-LSI at 60°/s was fixed at 65% according to the Youden index (Figure 2A). The ROC curve area for the H-LSI at 180°/s was 0.649 [95%CI: 0.595–0.704] and the sensitivity and specificity were, respectively, 62.8% and 54.3% if the cutoff of the H-LSI at 180°/s was fixed at 80% according to the Youden index.

#### 3.3.2. Second Model Taking into Account Limbs (Operated/Contralateral) as Unit of Analysis

First, the model included QS/BW at 60°/s on an operated limb with a correct prediction of 72.8% of patients (Hazard classification of 63.3%). The second step included QS/BW at 60°/s on the contralateral limb to achieve a prediction of 81.1% after the exclusion of the Lysholm score, knee complications, and H/Q ratios of the two limbs (Table 5). The *B* value of this second relevant parameter had a negative meaning; a lower value of QS/BW at 60°/s on a contralateral limb was associated with a higher rate of an RTR.

The ROC curve area for QS/BW at 60°/s on the operated side was 0.771 (95CI%: 0.725–0.818) and the sensitivity and specificity were, respectively, 69.3% and 70.2% if the cutoff was fixed at 1.60 Nm/kg according to the Youden index (Figure 2B). The ROC curve area for the QS/BW at 60°/s on a contralateral limb was 0.549 [95%CI: 0.492–0.607] and the sensitivity and specificity were, respectively, 58.2% and 48% if the cutoff was fixed at 2.50 Nm/kg according to the Youden index.

## 4. Discussion

In sport traumatology, clinicians are frequently faced with challenges such as a patient’s desire for a prompt return to the pre-injury level of practice as well as the occurrence of complications such as knee pain, swelling, and iterative rupture. An optimized rehabilitation protocol requires a step-by-step approach guided by valid criteria, including a return to running, to safely increase the overall proportion of the return to the expected level of performance.

This study showed a highly significant association between an RTR and isokinetic parameters, which is consistent with previous studies [15,16]. The quadriceps LSI at 60°/s was found to be the most accurate parameter to predict a return to running, with a cutoff of 65% associated with good sensitivity and specificity. The identification of this cutoff usefully fits into the clinical decision-making process to allow a return to running, which aims to increase the overall proportion of the return to the expected level of performance. The functional Lysholm score and knee complications were excluded during the forward selection of the predictive model because of their strong association with the isokinetic strength parameters in patients who had returned to running. Indeed, complications such as arthrofibrosis are known to be associated with a worse Q-LSI of 38% and a worse rate of a return to running (7%) [25]. In addition, at the time of the return to unrestricted physical activities, a strong correlation exists between knee joint function reported by the patients and quadriceps strength, the quadriceps LSI, and knee pain [26,27].

Quadriceps muscle weakness is a well-known phenomenon following knee trauma, surgery, or arthritis, mostly due to the arthrogenic muscle inhibition [28]. Arthrogenic muscle inhibition is a reflex inhibition of the musculature surrounding a joint after distension or damage to the structures of the knee joint, which has been linked to articular swelling, inflammation, and pain [28]. Strength deficit may persist for more than 6 months after ACLR, whereas no clinical signs of inflammation, such as pain, swelling, heat, and edema, can be found. A recent and unique study compared the running biomechanics of athletes before an ACL injury and after ACLR [29]. A deficit of the peak knee flexion angle and knee extensor moment in the operated limb persisted 12 months after ACLR [29]. A maximal knee extensor deficit (−57%) was detected 4 months after an ACLR when paradoxically a majority of athletes had already returned to running [14]. The kinematic and strength deficits reported on the operated limb led to altered gait patterns and compensations between limbs while walking, running, or squatting [29,30,31,32]. In addition, the symmetry of the gait pattern and quadriceps strength proved to be associated with a return to sport [33,34], highlighting the importance of evaluating limb symmetry indexes 4 months after ACLR.

Previous literature has questioned the usefulness of the Limb Symmetry Index to evaluate the ability to return to running and sport, regarding the strength deficit existing in the non-operated side after ACLR, even at 24 months post-surgery [35,36,37]. This contralateral strength deficit may lead to an overestimation of strength recovery on the operated side [38]. Therefore, we also analyzed the quadriceps strength-to-body-weight ratio (QS/BW) to evaluate whether this parameter is more appropriate than the Q-LSI. Furthermore, we performed two predictive models, one analyzing patients as a unit (LSI), and the other analyzing the limbs as a unit (strength reported to body weight). These two models revealed very similar percentages of correct predictions, 81.3% and 81.1%, respectively. Nevertheless, the second model achieved only 72.8% of the correct prediction with QS/BW on the operated side after the first step. Inclusion of the QS/BW on the contralateral side was necessary to achieve an 81.1% prediction, and the strength on the contralateral limb was inversely correlated with an RTR. Consequently, a lower difference of strength between the sides (higher strength on the operated limb and lower strength on the contralateral limb) increased the prediction of the RTR rate. We had to analyze the strength on both sides to more accurately predict the RTR. So, by directly using the Q-LSI, it is easier to allow a patient to return to running.

The H-LSI at 180°/s helps predict the RTR status. At 4 months post-surgery, the H-LSI is related to the recovery of hamstring strength [39]. This recovery can sometimes be late due to posterior knee pain or arthrofibrosis and is rarely complete at 12 months post-surgery (H-LSI: 83 to 95%) due to the regeneration of the hamstring tendons [40,41]. 

Interestingly, another study investigated the quadriceps strength-to-body-weight ratio (QS/BW) as an indicator of the RTR ability [16]. Using ROC curves, they found QS/BW to be significantly associated with a return to running with a cutoff of 1.45 Nm/kg (sensitivity 88.6%, specificity 87.2%), whereas Q-LSI was barely significant in multivariate regression (OR = 0.87; CI95% 0.75–1.00; *p* = 0.05). In our study, QS/BW showed a higher cutoff value of 1.60 Nm/kg. This apparent discrepancy may be explained by a delay in test completion at 3 months post-surgery in a study by Iwame et al. [16] versus the 4 months in ours. A strength deficit often persists for more than 3 months post-surgery, so it is expected that the QS/BW increases between the 3rd and 4th months after surgery. These different delays may explain the lower rate of a return to running of 53% [16] compared to 63.2% in our study. It would have been interesting to know the proportion of complications (pain, effusion …) that occurred among patients who failed the jogging trial to evaluate whether the delay of 3 months was premature or not.

In our study, a return to running was considered successful if patients had completed at least 50% of a running program, whereas only a jogging trial was performed in the other study [16]. Our method of evaluation is concrete, since complications such as pain or effusion may not occur during the first jogging session. On the other hand, we had to make a decision after the completion of the clinical (Lysholm score) and isokinetic tests whether or not to allow patients to attempt to return to running, which may have altered the outcome; patients who were allowed to attempt to return to running were more likely to do it, and vice versa. In this regard, we deemed it unethical to not take into account the isokinetic parameters in the decision-making process regarding evidences in the literature [14,15,16].

We included adult patients with primary ACLR using a hamstring autograft procedure. So, our results cannot be generalized to children, revision surgeries, and BPTB procedures, which require specific studies.

Another limit of our study is that the return to running depends on other parameters rather than functional measures and strengths such as psychological readiness and a patient’s motivation [7,42]. According to the patients, reasons not to return to sport are numerous: fear of re-injury, impaired knee function, instability, knee effusion, pain, muscle weakness, knee extension deficit, social reasons, and a lack of motivation. Hence, a return to running requires perhaps a higher multimodal approach that needs to be individually performed, and cannot be restricted to isokinetic tests [7,42,43]. Indeed, isokinetic assessment cannot account for social or psychological barriers to an RTR. However, knowing that Q-LSI at 60°/s and H-LSI at 180°/s can predict an RTR may be useful for preventing a premature return to running. Furthermore, we may be able to reassure patients who fear re-injury of their ability to return to running if the isokinetic parameters are greater than the cutoffs. This objective clinical approach is important and new because psychological readiness greatly alters an RTR [6,42,44]. A weakness of the study is that it is not possible to apply a standard protocol to all people in a rehabilitation program after ACLR. It should be adapted according to the clinical evolution of the knee and the patient’s objectives.

The next step would be to systematically and prospectively use these isokinetic cutoffs in patients who have primarily sustained ACLR associated with clinical tests as recommended [14]. The aim would be to evaluate the ability to safely increase the overall proportion of a return to running after ACLR with a hamstring autograft procedure. An isokinetic evaluation 4 months after surgery should be included in a standardized rehabilitation protocol and should be delayed if a clinical examination detects knee swelling, knee locking, extension loss > 15°, or a limp. The decision to perform an isokinetic evaluation sooner than at 4 months depends on the clinician’s experience, and first requires a full clinical recovery of the knee.

## 5. Conclusions

The best isokinetic parameter to help the decision-making process to return to running is the quadriceps LSI at 60°/s with a cutoff of 65% 4 months after ACLR with a hamstring procedure. The hamstring LSI at 180°/s can be added (cutoff of 80%) to slightly increase the certainty of the patient’s possibility of running. The quadriceps strength peak torque reported to body weight at 60°/s is also a useful parameter but should be measured on both sides to accurately predict a return to running (cutoff value of 1.60 Nm/kg for the operated side and of 2.50 Nm/kg for the non-operated side). It would be interesting to develop new, predictive isokinetic models in the future, including other functional and psychosocial parameters, to allow a greater number of patients to return to running and avoid knee complications after ACLR with a hamstring autograft procedure.

## Figures and Tables

**Table 1:** Comparison between the return-to-running and the no-return-to-running groups.

	Total Population *n* = 413	RTR *n* = 261	NRTR *n* = 152	*p*
Sex, *n* (%)				0.824 *
- Female	123 (29.8)	79	44	
- Male	290 (70.2)	182	108	
Mean age, years ± SD	26.2 ± 6.3	26.1 ± 6.4	26.4 ± 6.2	0.677
Mean weight, kg ± SD	73.0 ±13.0	73.3 ± 13.6	72.6 ± 11.9	0.618
Mean height, cm ± SD	173.9 ± 9.0	173.9 ± 10.0	173.9 ± 7.7	0.963
Delay ACL tear to surgery, days ± SD	197.2 ± 295.5	212 ± 284	173 ± 317	0.206
Delay surgery to isokinetic test, days ± SD	124.9 ± 20.2	125 ± 20.8	124 ± 19.3	0.899
Meniscal procedure, *n* (%)				0.522 *
- MM	50 (12.1)	31 (11.9%)	18 (11.8%)	
- LM	39 (9.5)	24 (9.1%)	15 (9.9%)	
- MM + LM	15 (3.6)	8 (3.1%)	8 (5.3%)	
- total	104 (25.2)	63 (24.1)	41 (27.0)	
No meniscal procedure	309 (74.8)	198 (75.9)	111 (73.0)	
Type of reeducation, *n* (%)				0.218 *
- Hospital	239 (57.9)	157 (60.2)	82 (53.9)	
- Ambulatory care	174 (42.1)	104 (39.8)	70 (46.1)	

* χ^2^ test. RTR: Return to Running; NRTR: No-Return to Running; SD: Standard Deviation; ACL: Anterior Cruciate Ligament; MM: Medial Meniscus; LM: Lateral Meniscus.

**Table 2:** Comparison of clinical and isokinetic parameters between the return-to-running and the no-return-to-running groups at 4 months post-surgery.

	RTR	NRTR	Mean Difference	CI95%	*p*
Clinical features:					
Mean Lysholm score	94.40	89.50	−4.90	[−6.80; −3.00]	<0.001
Isokinetic parameters:					
QS/BW 60°/s					
- Operated limb	2.06	1.38	−0.68	[−0.81; −0.55]	<0.001
- Contralateral limb	2.75	2.59	−0.15	[−0.32; 0.02]	0.08
QS/BW 180°/s					
- Operated limb	1.44	1.04	−0.40	[−0.49; −0.31]	<0.001
- Contralateral limb	1.79	1.68	−0.10	[−0.21; 0.004]	0.060
HS/BW 60°/s/kg					
- Operated limb	1.25	1.05	−0.20	[−0.29; −0.11]	<0.001
- Contralateral limb	1.50	1.42	−0.08	[−0.17; 0.02]	0.114
HS/BW 180°/s/kg					
- Operated limb	0.93	0.79	−0.14	[−0.21; −0.08]	<0.001
- Contralateral limb	1.10	1.04	−0.06	[−0.13; 0.01]	0.098
Q-LSI 60°/s, %	74.80	53.60	−21.20	[−24.3; −18.2]	<0.001
Q-LSI 180°/s, %	80.30	62.40	−17.90	[−20.9; −14.8]	<0.001
H-LSI 60°/s, %	84.00	73.70	−10.30	[−13.3; −7.4]	<0.001
H-LSI 180°/s, %	85.20	75.50	−9.70	[−13.1; −6.3]	<0.001
H/Q 60°/s					
- Operated limb	0.63	0.82	0.19	[0.14–0.25]	<0.001
- Contralateral limb	0.55	0.55	0.00	[−0.02; 0.02]	0.933
H/Q 180°/s					
- Operated limb	0.67	0.79	0.13	[0.08; 0.17]	< 0.001
- Contralateral limb	0.62	0.63	0.00	[−0.02; 0.03]	0.668

QS/BW: Quadriceps Strength peak torque to Body Weight ratio; HS/BW: Hamstring Strength peak torque to Body Weight ratio; Q-LSI: Quadriceps Limb Symmetry Index; H-LSI: Hamstring Limb Symmetry Index; H/Q: Hamstring-to-Quadriceps ratio; RTR: Return to Running; NRTR: No-Return to Running.

**Table 3:** Proportions of patients with complications amongst the return-to-running and the no-return-to-running groups.

Complications	Total Population, *n* (%)	RTR, *n* (%)	NRTR, *n* (%)
None	274 (66.3)	205 (78.6) *	69 (45.4) *
Arthrofibrosis	40 (9.7)	6 (2.3) *	34 (22.4) *
Anterior knee pain	26 (6.3)	17 (6.5) *	26 (17.1) *
Postero-internal knee pain	39 (9.4)	25 (9.6)	14 (9.2)
Knee joint swelling	28 (6.8)	5 (1.9)	6 (3.9)
Knee Infection	6 (1.5)	3 (1.1)	3 (2.0)

* *p* < 0.05, χ^2^ test (6 conditions × 2 groups); RTR: Return to Running; NRTR: no RTR.

**Table 4:** Models of prediction of a return to running, analyzing patient as a unit (limb symmetry indexes).

Prediction	*B*	Wald	ORs	CI95%	*p*
Step 1:					
Q-LSI 60°/s	8.57	93.2	52057	[1291–49,627]	<0.001
Constant	−5.27	75.3	0.005		<0.001
Step 2:					
Q-LSI 60°/s	8.57	93.2	52057	[1291–49,627]	<0.001
H-LSI 180°/s	2.65	10.8	14.11	[2.90–68]	0.001
Constant	−7.13	67.0	0.001		<0.001

Q-LSI: Quadriceps Limb Symmetry Index; H-LSI: Hamstring Limb Symmetry Index.

**Table 5:** Models of prediction of a return to running, analyzing legs (operated/contralateral) as a unit (peak torque to body weight).

Prediction	*B*	Wald	ORs	CI95%	*p*
Step 1:					
QS/BW 60°/s on operated limb	1.63	64.0	5.1	[3.41–7.58]	<0.001
Constant	−2.19	41.3	0.1		<0.001
Step 2:					
QS/BW 60°/s on operated leg	1.63	64.0	5.1	[3.41–7.58]	<0.001
QS/BW 60°/s on contralateral limb	−2.25	54.8	0.1	[0.06–0.19]	0.001
Constant	−0.07	0.03	0.9		0.87

QS/BW: Quadriceps Strength peak torque to Body Weight.

## Data Availability

The data presented in this study are available on request from the corresponding author. The data are not publicly available due to ethical reasons.
